# Evaluation of the effect of hyperchloremia on the prognosis and mortality of medical intensive care patients: a single-center study

**DOI:** 10.55730/1300-0144.5510

**Published:** 2022-08-21

**Authors:** Habip YILMAZ, Başak ÇAKIR GÜNEY

**Affiliations:** 1 Department of Anesthesiology and Reanimation, Public Hospital Services Administration, Directorate of Health of İstanbul, İstanbul, Turkey; 2 Department of Internal Medicine, Sultan 2. Abdülhamid Han Training and Research Hospital, University of Health Sciences, İstanbul, Turkey

**Keywords:** Medical intensive care unit, hyperchloremia, pCO_2_, mortality

## Abstract

**Background/aim:**

While chloride (Cl) is the most abundant anion in the serum, it is unfortunately one of the most commonly disregarded laboratory test results routinely drawn upon admission into the medical intensive care unit (MICU). We aimed to investigate the relation between in-hospital mortality, different pathologies requiring admission to the MICU, serum Cl levels, and other biochemical tests in a tertiary center.

**Materials and methods:**

The prospective study included data from 373 patients admitted to the ICU of a tertiary care center between 2017 and 2019. Data of patients under 18, pregnant patients or patients who were in the MICU for under 48 h were excluded. Comorbidity status, complete blood count, biochemistry tests, and blood gas analysis results of all patients included in the study were collected and recorded. Univariate and multivariate analyses were performed with the obtained data.

**Results:**

Of the patients included in the study, 158 (42.4%) were discharged, and 215 (57.6%) died. In the receiver operator characteristics curve analysis performed to determine the discriminating power of Cl levels with a cut-off value of >98 mEq/L in relation to mortality, its sensitivity was found to be 84% and specificity 60%. According to Kaplan–Meier analysis results, mortality rate was higher (60% vs 46%) and survival time was lower (19.0 ± 1.46 vs. 23.0 ± 4.36 days; p = 0.035) in the patient group with high Cl levels compared to the patient group with normal or low Cl levels. In the Cox regression analysis, it was found that the survival time of the patients hospitalized in the MICU was associated with the variables of Cl, presence of cancer diagnosis and pCO_2_ (hazard ratio: 1.030 (1.008–1.049), 2.260 (1.451–3.500), and 1.020 (1.003–1.029); p < 0.05, respectively).

**Conclusion:**

Mortality in MICU patients were found to increase in association with higher Cl levels at admission, presence of cancer disease, and higher pCO_2_ levels. In addition, it should not be ignored that there may be an important relationship between renal failure and hyperchloremia in MICU patients.

## 1. Introduction

Chloride (Cl) is one of the most abundant electrolytes in human serum, and yet, unfortunately, it is one of the most commonly ignored [1]. Anomalies in the blood Cl levels are common in hospitalized patients, and both hypochloremia and hyperchloremia have been linked to higher in-hospital mortality in general medical intensive care unit (MICU) patients [2]. Hypochloremia is an independent predictor of diuretic responsiveness and predicts short- and long-term mortality in patients with heart failure [3]. The fact that serum Cl imbalances are linked to negative outcomes in MICU and cardiovascular disease patients demonstrates the relevance of serum Cl in normal physiology.

Many pathophysiologic processes affect serum Cl, which is important for maintaining osmotic pressure, acid-base disturbances, and management of renal function. Serum sodium (Na) and Cl levels are intimately linked in maintaining plasma electroneutrality, and changes in volume status and plasma tonicity usually result in parallel changes in serum Na and Cl levels [4].

A certain portion of MICU patients are admitted for acute kidney injuries (AKI). A previous study by Komaru et al. demonstrated that lower urinary Cl concentrations are associated with a higher mortality in AKI patients [5]. Other studies have associated hyperchloremia with increased mortality after abdominal surgical interventions [6], and even subarachnoid hemorrhages [7]. In addition, a recent study reporting that [8] early detection of hyperchloremia can improve the prognosis of patients with lung cancer is important evidence that hyperchloremia may aggravate the condition of accompanying diseases.

In his physicochemical approach to theorizing acid-base physiology, Stewart recognized Cl as the primary strong negative ion of plasma. Along with these strong negative ions, pCO_2_ and plasma proteins acting as weak acids make important contributions as three independent parameters in determining hydrogen ion concentration and pH. In understanding the dependent variables (HCO_3_-, [HA], [A-], [CO_2_], [OH-], and [H+] (or pH)) in a solution, the independent variables including Cl (pCO_2_, net strong ion charge, and total weak acid, usually protein) need to be known as the dependent variables that are determined by the independent variables [9]. Considering imbalances in acid-base status of these patients are usually the main reason for the ICU admission, in light of this approach, we theorized that the combination of acid-base status, comorbidity status, biochemical parameters and Cl concentration in evaluating the MICU patient’s survival could be worth more than the sum of its components alone. We investigated the relationship between patients’ mortality and different pathologies requiring admission to the MICU, serum Cl levels, and other biochemical parameters.

## 2. Materials and methods

### 2.1. Data collection

The study was designed as an analytical type retrospective research. Data for the consecutive patients who were admitted to Health Sciences University Sultan 2. Abdülhamid Han Training and Research Hospital Medical ICU between 1 January 2017 and 31 December 2019 were included in the study. The patients were either admitted directly to the ICU from the emergency service, or transferred to the ICU from the wards. Electronic medical records for the patients were analyzed for biochemistry panels, sex, age, diuretics use, 0.9% NaCl use, and prior cancer, kidney failure (acute or chronic), diabetes mellitus, septicemia, stroke, heart failure, pulmonary insufficiency, and myocardial infarction diagnosis. Patients who were under 18 years old, who were pregnant, or who were in the ICU for less than 48 h were excluded from the study. Ethical approval for the study protocol was given by the institutional review board of Süreyyapaşa Chest Diseases and Thoracic Surgery Training and Research Hospital, with protocol code 116.2017.R-245.

### 2.2. Laboratory analysis

The samples for Cl, pH, and other biochemistry measurements were drawn from the arterial line placed in the patients’ radial artery immediately upon admission. The Cl levels and other biochemistry parameters were measured using the Cobas 8000 c502 analyzer (Roche Diagnostics, Rotkreuz, Switzerland). Blood pH was measured using the ABL 700 Blood Gas Analyzer (Radiometer Medical, Copenhagen, Denmark).

### 2.3. Diagnoses

The diagnosis of diabetes mellitus was made in accordance with American Diabetes Association (ADA) diabetes criteria [10]. Septicemia was defined as the presence of bacteria in blood cultures routinely drawn at the time of admission, and symptoms compatible with bacteremia. Cancer diagnoses were either preexisting, with evidence in the electronic medical records, or diagnosed during the MICU stay.

### 2.4. Statistical analysis

Data analysis was performed on the RStudio version 2022.02.1 (RStudio PBC, Massachusetts, USA). Histogram and Shapiro–Wilk’s test were used to evaluate the conformity of continuous variables to normal distribution. The demographic data and characteristics of the study population, frequencies and percentages for categorical variables and means, standard deviation, and lower and upper limits for continuous variables were compiled at 95% confidence intervals. Statistical significance was examined using chi-square and analysis of variance F tests (overall comparisons) and trend tests (Mantel–Haenszel test for categorical variables and Spearman’s rank test for continuous variables). The Kaplan–Meier method was used for the relationship between Cl and mortality, and the log-rank test was used to evaluate the differences.

The relationship between Cl, mortality, cancer diagnosis, pCO_2_, and other independent variables was investigated using the Cox regression analysis. The optimal cut-off point for Cl levels associated with mortality was determined using the ROC and maximum Youden index (highest sensitivity + specificity).

Post hoc power analysis was used to estimate the strength of the observed effect based on the sample size and Cl parameter of our dataset. As a result of the analysis, the effect size d was found to be 0.29 and the power (1-β err prop) was 0.9165.

## 3. Results

**Figure 1:**
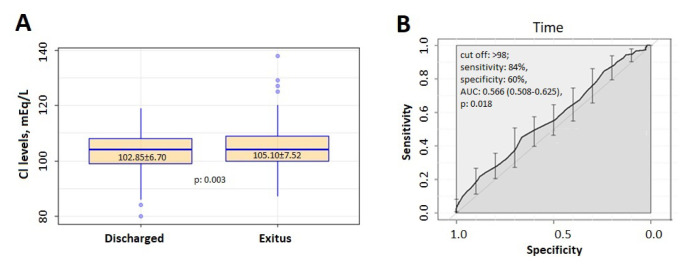
A) In the figure, it is seen that there is a statistical difference between the chlorine levels of the discharged and exitus patients. B) Receiver operating characteristic curve for chloride to predict mortality.

The study, covering the three-year period from 2017 to 2019, was composed of data from 373 patients—185 (%49.6) men and 188 (50.4) women—treated in the MICU. It was determined that 158 (42.4%) of the patients were discharged and 215 (57.6%) died. The mean age of the participants in the study was 74.39 ± 13.09 years, the length of stay in the MICU was 18.07 ± 19.59 days, and the Cl level was 104.67 ± 7.33 mEq/L. The length of stay in the MICU of those who were discharged was found to be statistically lower compared to the group who died (12.97 ± 17.49 vs. 21.82 ± 20.23; p < 0.0001). Cl levels of those who were discharged were statistically lower compared to the group who died (102.85 ± 6.70 vs. 105.10 ± 7.52 mEq/L; p = 0.003) (Figure 1A). In the ROC analysis for the mortality-related diagnostic value of hyperchloremia, the area under the curve (AUC) was statistically significant (AUC: 0.57 (0.51–0.63); p = 0.018). Mortality cut-off values from the area under the ROC (receiver operator characteristics curve) curve for Cl levels were calculated as >98 mEq/L (84% sensitivity, 60% specificity) (Figure 1B).

When Cl level cut-off values are taken as 98 mEq/L and classified as normal or high; chance of mortality (44% vs 61%), renal failure prevalence (27% vs 45%), urea (89.09 ± 56.57 vs 121.35 ± 65.27 mg/dL), Na (133.87 ± 7.32 vs 140.34 ± 7.94 mEq/L), C-reactive protein (CRP) 86.44 ± 67.27 and 126.26 ± 92.7 mg/L), and partial pressure of carbon dioxide (pCO_2_) (47.16 ± 13.47 vs 40.88 ± 12.41 mmHg) were found to be correlated (p < 0.05) (Table 1). There was no statistical difference between the groups in term of sex, age, diuretics use, 0.9% NaCl use, and prior cancer, kidney failure, diabetes mellitus, septicemia, stroke, heart failure, pulmonary insufficiency, and myocardial infarction diagnosis.

**Figure 2:**
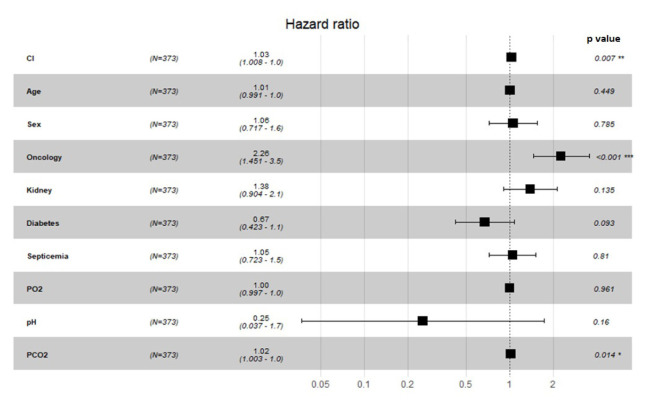
Forest plot depicting the association between characteristics of study patients and mortality in model (Cox regression).

A logistic regression model was created with all of the study variables (Cl, sex, age, cancer, renal failure, diabetes, septicemia, pO_2_, pH, pCO_2_, urea, Na, and CRP) that are thought to affect mortality in intensive care unit patients (p < 0.05, Hosmer and Lemeshow test). Stroke, heart failure, pulmonary insufficiency, and myocardial infarction were not included in the logistic regression model because the number of cases was very low. As a result of the forward technique applied; high Cl level [HR: 1.030 (1.008–1.049), p = 0.013], presence of cancer diagnosis (HR: 2.260 (1.451–3.500), p < 0.0001) and pCO_2_ level (HR: 1.020 (1.003–1.029), p=0.002) were found as variables affecting mortality (Figure 2 and Table 2). pO_2_ and pH were found to be statistically ineffective on mortality in intensive care unit patients (p > 0.05).

**Figure 3:**
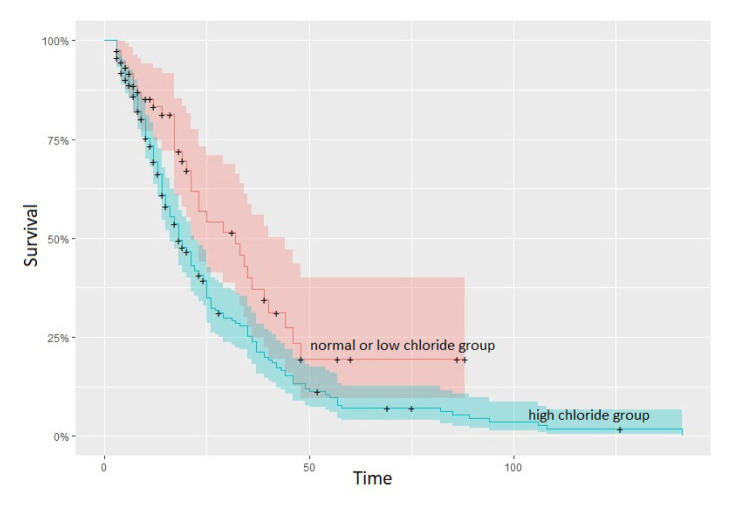
Kaplan–Meier Survival Function Curve of Association Between Chloride Levels and Mortality.

The mean survival time of the patients participating in this study was 21.00 ± 1.43 (18.19–23.81) days. According to Kaplan-Meier analysis results, mortality rate was higher (60% vs 46%) and survival time was lower (19.0 ± 1.46 vs. 23.0 ± 4.36 days; p = 0.035) in the patient group with high Cl levels compared to the patient group with normal or lower Cl levels (Figure 3).

The relationship between Cl and cations in the study data was examined. While there was a statistically significant positive correlation between Cl and Na (Spearman r = 0.61; p < 0.0001), no correlation was found between Cl and K^+^ and Mg^+2^ (p > 0.05). In addition, there was a weak but statistically significant negative correlation between Cl and pCO_2_ levels (Spearman r = −0.201, p = 0.003).

## 4. Discussion

The MICU is a place that caters to a wide array of nonsurgical pathologies. Serum electrolyte assays are a point of focus in the care of ICU patients, and their follow-up directly influences the acute management of several clinical scenarios including the disease management of respiratory, cardiovascular, renal, and gastrointestinal systems among several others. These systems are closely interlocked with each other, and these relations often manifest through the changes they make in the blood chemistry, such as electrolytes (Na, K, and Cl) and blood gas. Except for blood Cl levels, many biological markers have been associated with quite different diseases and are widely used in MICU admission and follow-up. However, Cl constitutes an important part of the negative ion content of the body. It also has an important role in maintaining the osmolarity, acid-base balance, and electro-neutrality of body fluids [11–13]. Unfortunately, there is insufficient information about the relationship between blood Cl levels and MICU admission and mortality.

In this analysis, we analyzed the data of 373 patients and concluded that there is an inverse relation between Cl levels and survival chance of an MICU patient. To our knowledge, this is one of the few studies in the literature that included only MICU patients in its cohort. This may help highlight the role Cl plays in management of nonsurgical pathologies in the MICU. The most important finding of our study was that Cl levels above our threshold of 98 mEq/L are associated with increased mortality. This finding could potentially have multiple explanations. Death in MICU patients often occur through cardiac arrests, which are more difficult to resuscitate because most dying patients have abnormal electrolyte and homeostatic disturbances that predispose to cardiac arrest before the event [14].

Sepsis and septic shock are one of the leading causes of mortality in the MICU [15]. Hypotension is a major feature of septic shock, and the ICU physician is inclined to infuse crystalloids to increase blood pressure in such patients. A significant portion of septic shock patients respond to fluid resuscitation to an extent [16], and appropriate fluid resuscitation with balanced fluids or albumin has been demonstrated in previous studies to reduce mortality in septic shock [17]. The absence of a relationship between septicemia and hyperchloremia or mortality rate in our MICU patient group was also attributed to the good response of septicemia to drug therapy and fluid replacement. Moreover, the fact that only 33% of our study group had septicemia may have been reflected in the results.

Saline is commonly defined as an isotonic NaCl solution in water. While it is indeed isotonic to blood [18], its NaCl concentration is higher than blood. In 2018, the SMART trial conducted by Semler et al. [19] demonstrated that fluid resuscitation with balanced fluids were associated with lower deaths from any cause, lower emergent need for renal replacement therapy, or lower acquired renal dysfunction than normal saline. One of the explanations for this phenomenon is that the higher Na and Cl content leads to hyperactivation of the renin–angiotensin–aldosterone system, and desreases renal cortical perfusion and intracapsular hypertension. This may bring up the idea of iatrogenic hyperchloremic metabolic acidosis, contributing to mortality in MICU patients, but a 2017 study by Yessayan et al. [20] demonstrated that hyperchloremia developed after MICU admission was not associated with an increased risk for AKI at 72 h. On the contrary, Suetrong et al. [21] found that while higher baseline Cl levels at admission are associated with higher AKI in sepsis patients, increases in serum Cl during the MICU stay are also associated with increased risk of AKI, even if the patient’s baseline Cl level was within normal range. A 2012 study by Yunos et al. [22] also confirms the results obtained by Suetrong et al. The results of this study seem to be compatible with the study results obtained by Suetrong and Yunos.

As could be expected by the pathophysiology, our study found that serum Cl levels were higher in patients with chronic renal failure. As Robert Luke discussed in his 1979 article [23], in chronic renal failure, there is an increase in Cl reabsorption along the tubule in response to increased glomerular excretion of unreabsorbable anions such as phosphates and sulfates. While it may not be of concern for the MICU physician who usually follows their patients for a relatively short amount of time, in 2020, Khatri et al. [24] demonstrated that a higher serum Cl level is associated with a statistically significantly faster decline in renal function in chronic renal failure patients. All these findings explain why we found higher levels of Cl in people with kidney failure in this study. In addition, the detection of high Na and urea levels in patients with hyperchloremia also supports these findings.

Another significant finding of our study was the correlation between serum Cl levels and pCO_2_. While pCO_2_ in itself plays a major role in the acid base balance, it is also affected by the role Cl plays as a strong anion and its contribution to the strong ion difference. An increase in the serum Cl may be decreasing HCO_3_ level and therefore increasing CO_2_ levels by way of the Henderson–Hasselbalch equation [25].

Hyperchloremia in cancer patients admitted to the MICU was significantly associated with increased mortality. This could be due to a higher basal Cl level arising from the increased cell turnover and necrosis of neoplastic tissue. The aggravation in cell necrosis caused by the primary pathology leading to ICU admission could further increase this basal Cl level [8].

In our study, hospital stays were statistically significantly longer in patients with hyperchloremia. A few scenarios for this may be at play in getting this result. Improper fluid resuscitation in patients with renal or heart failure may lead to fluid overload, resulting in longer hospital stays to correct the acquired volume overload. Similarly, Cl rich fluid replacement in septic patients increases the risk for an AKI and may lead to an increase in the need for renal replacement therapy, which can last days before the patient is able to be weaned off of it. This contributes to the length of the hospital stay.

While this study did not analyze the iatrogenic hyperchloremia specifically, the adverse outcomes associated with hyperchloremia at the time of admission may suggest avoiding the development of iatrogenic hyperchloremia.

Our study had a few limitations. Firstly, it was conducted in a single center; therefore, the results may not be extended to the universe. Secondly, the residual selection bias may still be present while data of consecutive patients was recruited to the study. Third confounding factors may still apply to the results.

## 5. Conclusion

Mortality in MICU patients were found to increase in association with higher Cl levels at admission, presence of cancer disease, and higher pCO_2_ levels. In addition, it should not be ignored that there may be an important relationship between renal failure and hyperchloremia in MICU patients. Although no significant effects on mortality were detected in Cox regression analysis, urea and Na levels should not be ignored in MICU follow-up due to their association with hypo-/hyperchloremia and renal failure.

## Figures and Tables

**Table 1:** Characteristics of study patients by chlorine level.

	n	Hypochloremia 77	Hyperchloremia 296	p-value
Status (ex), n	215	34 (44%)	181 (61%)	^a^0.007*
Sex (male), n(%)	188	38 (49%)	150 (51%)	^a^0.84
Cancer, n(%)	88	18 (23%)	71 (24%)	^a^0.91
Kidney failure, n	154	21 (27%)	133 (45%)	^a^0.005*
Diabetes mellitus, n(%)	71	13 (17%)	58 (20%)	^a^0.59
Septicemia, n(%)	122	20 (26%)	102 (34%)	^a^0.16
Stroke, n(%)	20	5 (7%)	15(5%)	^a^ 0.62
Heart failure, n(%)	57	17 (22%)	40(14%)	^a^0.075
Pulmonary insufficiency, n(%)	8	1(1%)	7(2%)	^b^0.99
Myocardial infarction, n(%)	7	2(3%)	5(2%)	^b^0.64
Diuretics use	76	14(18%)	62(21%)	^a^0.59
0.9% NaCl use	373	77	296	^-^
Age, year		71.36 ± 13.82	75.18 ± 12.8	^d^0.03*
Time, day		20.05 ± 18.19	17.56 ± 19.93	^c^0.32
Urea, mg/dL		89.09 ± 56.57	121.35 ± 65.27	^c^ 0.0001*
Na, mEq/L		133.87 ± 7.32	140.34 ± 7.94	^d^ 0.0001*
Mg, mg/dL		1.83 ± 0.49	1.89 ± 0.59	^c^ 0.63
K, mEq/L		3.94 ± 0.94	4.2 ± 1.03	^c^ 0.12
CRP, mg/L		86.44 ± 67.27	126.26 ± 92.7	^d^ 0.001*
pO_2_, mmHg		77.36 ± 44.13	83.15 ± 59.8	^d^ 0.49
pH		7.38 ± 0.11	7.38 ± 0.1	^d^ 0.81
pCO_2,_ mmHg		47.16 ± 13.47	40.88 ± 12.41	^d^ 0.001*

Overall comparison with (a) chi-squared test and (b) Fisher’s exact test for categorical variables, (c) t-test for parametric variables and (d) Mann–Whitney U Test for nonparametric variables.

* Statistically significant, Ex: exitus, Diuretics use: thiazide, loop, or potassium-sparing diuretics use, NaCl: sodium chloride.

**Table 2:** Multivariate Cox regression analysis to determine the independent variables affecting mortality.

Variables	B	Exp(B)	95% CI Lower–upper	p
Cl	0.03	1.030	1.008–1.049	0.013*
Age	0.01	1.010	0.991–1.000	0.138
Sex	0.01	1.060	0.717–1.600	0.596
Cancer	0.39	2.260	1.451–3.500	<0.0001*
Kidney failure	0.21	1.380	0.904–2.100	0.061
Diabetes mellitus	–0.26	0.670	0.423–1.100	0.178
Septicemia	–0.18	1.050	0.723–1.500	0.649
pO_2_	0.00	1.000	0.997–1.000	0.378
pH	–2.16	0.250	0.037–1.700	0.611
pCO_2_	0.01	1.020	1.003–1.029	0.002*
Urea	–0.001	0.999	0.996–1.002	0.392
Na	–0.016	0.984	0.960–1.008	0.104
CRP	0.002	1.002	1.000–1.005	0.071

* Statistically significant, Cl: chloride, pO_2_: partial pressure of oxygen, pCO_2_: partial pressure of carbon dioxide, pH: hydrogen ion concentration, CI: confidence interval, Na: sodium, CRP: C-reactive protein
